# CAFLOW: Conditional Autoregressive Flows

**DOI:** 10.3934/fods.2024028

**Published:** 2024

**Authors:** Georgios Batzolis, Marcello Carioni, Christian Etmann, Soroosh Afyouni, Zoe Kourtzi, Carola-Bibiane Schönlieb

**Affiliations:** DAMTP, University of Cambridge, Cambridge CB3 0WA; Department of Psychology, University of Cambridge, Cambridge CB2 3EB; DAMTP, University of Cambridge, Cambridge CB3 0WA

## Abstract

We introduce CAFLOW, a new diverse image-to-image translation model that simultaneously leverages the power of auto-regressive modeling and the modeling efficiency of conditional normalizing flows. We transform the conditioning image into a sequence of latent encodings using a multi-scale normalizing flow and repeat the process for the conditioned image. We model the conditional distribution of the latent encodings by modeling the auto-regressive distributions with an efficient multi-scale normalizing flow, where each conditioning factor affects image synthesis at its respective resolution scale. Our proposed framework performs well on a range of image-to-image translation tasks. It outperforms former designs of conditional flows because of its expressive auto-regressive structure.

## 1 Introduction

Generative modeling has emerged as one of the most widely-researched areas in deep learning over the last few years. While generative adversarial networks (GANs) [11] produce state-of-the-art results for images [38], they do not allow for the estimation of likelihoods. Other types of generative methods, however, admit the explicit evaluation of likelihoods of points under the given model, and thus allow for training as maximum likelihood estimators. Normalizing flows [33] (and their continuous formulation, via NeuralODEs [7]) are one such example. These *flow-based* models are diffeomorphic neural networks, which are trained to invertibly transform data into e.g. a normal distribution. This is made possible by a change-of-variables formula, which expresses the likelihood of a data sample in terms of the normal distribution. Samples from the approximated distribution are then generated by passing samples from a normal distribution through the inverse function. A straightforward extension of likelihood estimators are *conditional* likelihood estimators, which represent the likelihood of a random variable conditioned on another random variable. This is in particular used in autoregressive models and their flow-based variants (*autoregressive flows* [15]). These types of models explicitly parametrize joint likelihoods via the product rule for probability densities, and can hence be used for creating expressive likelihood estimators, at the cost of more involved computations. This approach is in particular employed by Wavelet Flows [44], which use a hierarchical multi-scale representation via Wavelet decompositions in order to parametrize expressive conditional likelihood functions for images.

Domain transfer, e.g. via models such as CycleGAN [46] and DUAL-GLOW [37], is another topic that has received wide interest in recent years. In domain transfer, the task is to *transfer* a data point from some *origin* domain (e.g. black-and-white images) to a different *target* domain (color images in this example). The technique of conditioning lends itself well to domain transfer, as it is possible to condition likelihood functions over target domain on points from the origin domain, thereby making conditional generation possible. In flow-based models, this has been introduced in the form of conditional flows [40], which function based on this general principle. SRFlow [27] and DUAL-GLOW can be seen as special cases of this, where the conditioning on the origin domain is done in a multi-scale fashion.

### 1.1 Contributions

In this work, we introduce CAFLOW, *conditional autoregressive flows*, which combine the idea of conditional flows with the expressiveness of autoregressive models using hierarchical multi-scale flows for domain transfer. As a first step, our model encodes the conditioning and the conditioned images into a hierarchical sequence of latent spaces. Then, using an autoregressive approach, it decomposes the conditional distribution of the latent encodings into *n* autoregressive components with the aim of modeling each component using a conditional normalizing flow. By carefully designing such a flow based on weak assumptions on the mutual dependencies of the latent encodings, our model allows for exchange of information between latent spaces of different dimension. In particular, our designed architecture is able to capture correlations between different scales, improving on the expressivity of other conditional flow-based models used for domain transfer tasks. To the best of our knowledge, our work is the first to explore cross-scales correlations in the context of conditional normalizing flows for domain adaptation.

Our modeling choices are corroborated by strong experimental evidence. We demonstrate that our model achieves good results on classical image-to-image translation tasks such as image super-resolution, image colorization and image inpainting. In particular, we show that on these tasks CAFLOW outperforms former designs of conditional flows and GAN-based models thanks to its expressive auto-regressive structure.

## 2 Background

### 2.1 Normalizing flows

Given a random variable *Y* with an unknown distribution *p*_*Y*_, the main idea of flow-based generative modeling is to approximate *p*_*Y*_ with a learned distribution $p_{Y}^{\theta }$, which is parametrized by an invertible neural network *G*^*θ*^ mapping from a latent space *Z* to *Y*. Choosing *p*_*Z*_ to be a tractable distribution on *Z*, we define the distribution $p_{Y}^{\theta }$ as the one obtained by applying the mapping ***y*** = *G*^*θ*^(***z***) to the distribution *p*_*Z*_. By the change of variables formula for probability distributions, $p_{Y}^{\theta }$ is easily computed as

$$p_{Y}^{\theta }(y)=p_{Z}\left(F^{\theta }(y)\right)\left|\det\left(\frac{dF^{\theta }(\text{y})}{d\text{y}}\right)\right|,$$

where *F*^*θ*^ is the inverse of *G*^*θ*^. Thanks to the formula above it is then possible to match $p_{Y}^{\theta }$ to *p*_*Y*_ by miminizing the negative log-likelihood of $p_{Y}^{\theta }$,

$$-\log p_{Y}^{\theta }(y)=-\log\left(p_{Z}\left(F^{\theta }(y)\right)\right)-\log\left|\det\left(\frac{dF^{\theta }(y)}{dy}\right)\right|.$$

In practice, the transformation *G*^*θ*^ is a composition of learnable invertible transformations *g*_1_, …, *g*_*n*_ such that *g*_*i*_ : *Z*_*i*_ → *Z*_*i*+1_ (where *Z*_1_ = *Z* and *Z*_*n*+1_ = *Y*) for intermediate latent spaces *Z*_*i*_ and

(1) $$y=g_{n}\left(g_{n-1}\left(\ldots g_{1}(z)\right)\right)=G^{\theta }(z),\;z=f_{1}\left(f_{2}\left(\ldots f_{n}(y)\right)\right)=F^{\theta }(y),$$

where *f*_*i*_ = (*g*_*i*_)^−1^ for every *i* and thus *F*^*θ*^ = (*G*^*θ*^)^−1^. This model is called a normalizing flow. In this case, using the properties of the Jacobian for composition of invertible transformations the log-likelihood of $p_{Y}^{\theta }$ can be computed as

(2) $$\log\left(p_{Y}^{\theta }(y)\right)=\log\left(p_{Z}\left(F^{\theta }(y)\right)\right)+\sum_{i=1}^{n}\log\left|\det\left(\frac{df_{i}\left(z_{i+1}\right)}{dz_{i+1}}\right)\right|,$$

where ***z***_*n*+1_ = ***y***. In order to estimate log $\left(p_{Y}^{\theta }(y)\right)$ the transformations *g*_*i*_ need to be designed to have a computable inverse *f*_*i*_ and a tractable Jacobian determinant. We next describe typical choices for the transformations *g*_*i*_ introduced in [20, 8] and then commonly used in most of the normalizing flow architectures. We will refer to them in the next sections where we describe our method.

#### Affine coupling layer

Affine coupling layers [8] capture complex dependencies of the activations in an invertible way. They split the activation in the dimension of the channels creating two components ***z***_1_ and ***z***_2_. ***z***_2_ is untouched by the transformation and is used to calculate the scale and the bias in the affine transformation of ***z***_1_ as shown in (3). The scale and the bias are calculated by parametrized neural networks *h*_*s*_ and *h*_*b*_ which do not need to be invertible.

(3) $$\begin{array}{l} y_{1}=\exp\left(h_{s}\left(z_{2}\right)\right)z_{1}+h_{b}\left(z_{2}\right)\\ y_{2}=z_{2} \end{array}$$

The output is then concatenated in [***y***_1_, ***y***_2_]. Note that the inverse and the Jacobian determinant of the transformation (3) can be efficiently computed [8].

#### Invertible 1 × 1 convolution

Invertible 1 × 1 convolutions have been introduced in GLOW [20]. They replace standard convolutional layer for which inverse and Jacobian determinant are not tractable. They mix the activations in the dimension of the channels so that different parts of the activation are modified by subsequent coupling layers.

#### Actnorm

Actnorm has been introduced in [20] as a variant of the classical batch normalization [16] where the minibatch size is equal to one. It performs an affine transformation using a scale and bias parameter per channel. It improves the training stability.

### 2.2 Conditional Normalizing Flows

The normalizing flow approach can be adapted to model conditional densities of complicated target distributions. Precisely, a conditional density *p*_*Y* |*X*_ is parametrized using a transformation *G*^*θ*^ : *Z* × *X* → *Y* such that *G*^*θ*^(·, ***x***) : *Z* → *Y* is invertible for every condition ***x***. We denote by $p_{Y|X}^{\theta }$ the distribution obtained by applying the mapping ***y*** = *G*^*θ*^(***z, x***) to samples ***z*** from a simple distribution *p*_*Z*_. By the change of variables formula for probability distributions the conditional log-likelihood of $p_{Y|X}^{\theta }$ can be then computed as

(4) $$\log\left(p_{Y|X}^{\theta }(y|x)\right)=\log\left(p_{Z}\left(F^{\theta }(y,x)\right)\right)+\log\left|\det\left(\frac{\partial F^{\theta }(y,x)}{dy}\right)\right|,$$

where *F*^*θ*^(***y, x***) is the inverse of *G*^*θ*^(***z, x***) for every condition ***x***. Consequently, a generative model for *p*_*Y*|*X*_ can be trained by minimizing the negative log-likelihood of the parameters *θ* using the formula in (4). The sampling procedure works similarly to standard normalizing flows. We generate a sample *y* ~ *p*_*Y* |*X*_ by sampling a latent ***z*** ~ *p*_*Z*_ and passing it through *G*^*θ*^(·, ***x***), yielding *y* = *G*^*θ*^(*z*, ***x***). Similarly to traditional normalizing flows the map *G*^*θ*^ is modelled through a composition of learnable invertible conditional transformations. We describe next the specific layers used in conditional normalizing flow architectures.

#### Conditional affine coupling layer

It is a conditional variant of the affine coupling layer. The difference is that the scale and the bias are calculated by parametrized functions of both the slit activation ***z***_2_ and the condition ***x*** as shown in (5).

(5) $$\begin{array}{l} y_{1}=\exp\left(h_{s}\left(z_{2},x\right)\right)z_{1}+h_{b}\left(z_{2},x\right)\\ y_{2}=z_{2} \end{array}$$

#### Affine Injector

The affine injector layer has been introduced in [27] to transfer more information from the conditioning data to the main branch of the flow. It is an affine transformation of the flow activation ***z***, where the scale and the bias are parametrized functions of the condition ***x***.

$$y=\exp\left(h_{s}(x)\right)z+h_{b}(x).$$

The inverse and the Jacobian determinant of such transformation can be easily computed as in [27].

## 3 Related work

Domain adaptation and image-to-image translation tasks have received a lot of attention in recent years, thanks to the development of representation learning methods [4] and the advent of adversarial approaches [11, 3]. A plethora of methods based on GANs have been proposed for solving super-resolution, colorization, inpainting and many other domain adaptation tasks [46, 5, 14, 6, 39, 28, 45, 24, 1] achieving remarkable results. Along with GAN-based models, other alternatives have been developed: Variational Autoencoders [19] approaches [42], score-based models [35] and more in general models that use specific discrepancies to measure the similarity between source and target distributions [34, 36, 28, 23, 31]. Conditional normalizing flows and their multi-scale variants have received much less attention for solving domain adaptation tasks. However, in recent years, they have gained popularity. Designed to match source and target distributions by maximizing the likelihood of a parametrized family of probabilities [20, 9, 40, 33] they have been used to achieve comparable performances to GAN-based models in super-resolution [27, 40], inpainting [26] and image-to-image translation [32, 37, 12]. Autoregressive models and autoregressive flows are considered one of the current state-of-the-art architecture for image modeling and they have been employed successfully for various tasks, such as image generation, image completion and density estimation [15, 29, 30, 22, 10, 21]

### 3.1 DUAL-GLOW

Here we describe in more detail the DUAL-GLOW model introduced in [37], as it shares some similarities with our approach. DUAL-GLOW proposes to couple two multi-scale normalizing flows modelled with a GLOW architecture that interact at different scales. Their main goal is to translate MRI images to PET images, but the same architecture has proven to be useful for other standard image-to-image translation tasks. They estimate the conditional probability *P* (*W*|*Y*) using two multi-scale normalizing flows which convert images *Y* and *W* into their respective hierarchical latent spaces [*D*_*n*−1_, …, *D*_0_] and [*L*_*n*−1_, …, *L*_0_], as shown in schematically in Figure 2. They model the conditional distribution *P* (*L*_*i*_|*D*_*i*_) by a parametrized multidimensional Gaussian distribution whose mean *µ*^*θ*^ and covariance Σ^*θ*^ are learnable functions of *D*_*i*_. Then they use *P* (*L*_*i*_|*D*_*i*_) to estimate *P* (*L*_*n*−1_, …, *L*_0_|*D*_*n*−1_, …, *D*_0_) under the implicit assumption that information is exchanged only between latent spaces of the same dimension, that is

(6) $$P\left(L_{n-1},\ldots ,L_{0}|D_{n-1},\ldots ,D_{0}\right)=\prod_{i=0}^{n-1}P\left(L_{i}|D_{i}\right).$$

## 4 Method

The aim of our CAFLOW model is to perform image-to-image translation tasks by learning the conditional distribution *P* (*W* | *Y*) where *W* and *Y* are random variables that model given image distributions. Following a similar architecture to [37] we use two multi-scale normalizing flows *R*^*θ*^ and *T*^*θ*^ to convert images *Y* and *W* into two sequences of *n* hierarchical latent spaces of decreasing dimension: $R^{\theta }(Y)={\tilde{D}}_{n}$, where ${\tilde{D}}_{n}≔\left[D_{n-1},\ldots ,D_{0}\right]$ and $T^{\theta }(W)={\tilde{L}}_{n}$, where ${\tilde{L}}_{n}≔\left[L_{n-1},\ldots ,L_{0}\right]$. Then we design an autoregressive model based on conditional normalizing flows to learn the conditional distribution $P\left({\tilde{L}}_{n}|{\tilde{D}}_{n}\right)$.

### 4.1 Modeling assumptions

**Figure 1:**
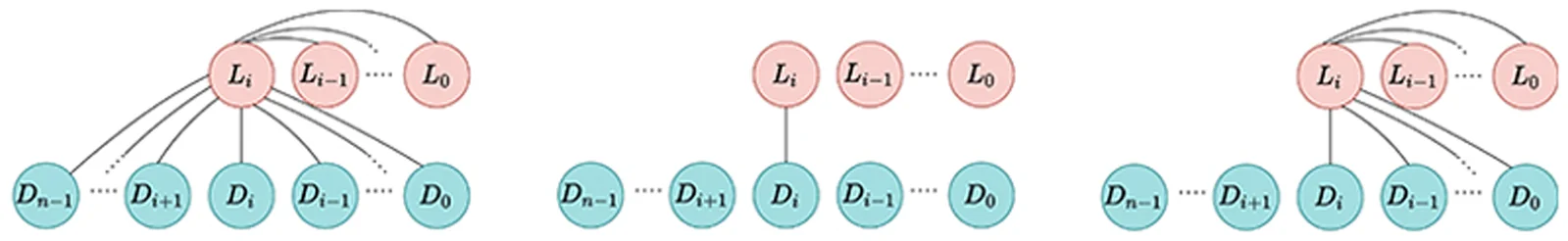
From left to right: ideal dependencies in the *i*^*th*^ autoregressive component. DUAL-GLOW modeling assumption [37]; information is exchanged only between latent spaces having the same dimension. Our modeling assumption; we retain the dependencies between *L*_*i*_ and the latent spaces of lower dimension.

The conditional distribution $P\left({\tilde{L}}_{n}|{\tilde{D}}_{n}\right)$ is factorized using the chain rule of probability into *n* autoregressive component distributions as shown in (7):

(7) $$P\left({\tilde{L}}_{n}|{\tilde{D}}_{n}\right)=\prod_{i=0}^{n-1}P\left(L_{i}|{\tilde{L}}_{i},D_{n-1},\ldots ,D_{0}\right)$$

with the notational convention that ${\tilde{L}}_{0}=\emptyset$ and ${\tilde{L}}_{i}=\left[L_{i-1},\ldots ,L_{0}\right]$ for every *i*. The dependencies of the *i*^*th*^ component distribution are shown graphically in the left diagram of Figure 1. Modeling those *n* autoregressive distributions can be unnecessarily computationally expensive. For this reason, we assume that

(8) $$P\left({\tilde{L}}_{n}|{\tilde{D}}_{n}\right)=\prod_{i=0}^{n-1}P\left(L_{i}|{\tilde{L}}_{i},{\tilde{D}}_{i+1}\right),$$

where ${\tilde{D}}_{i+1}=\left[D_{i},\ldots ,D_{0}\right]$. In particular, we retain the dependencies between *L*_*i*_ and all *L* and *D* latent variables of level *i* and below, which effectively means that we are pruning only the dependencies between *L*_*i*_ and *D*_*i*+1_, …, *D*_*n*−1_. We advocate that this is a valid assumption, because the encoded information in the split variables of the multi-scale flow typically ranges from local noise patterns and image details to higher-level information as we move from the early split variables to the final split variables. We remark that our modeling assumption is weaker than the one implicitly used in [37] described in (6) (see Figure 1). Precisely we allow information to be exchanged between latent spaces of different dimension. As we will demonstrate in the experiments our choice allows for more expressive architectures able to capture correlations between different scales. We represent schematically in Figure 1 the difference between the theoretical latent space dependencies, the one assumed in [37] and our modeling choice. Under this modeling assumption, our goal is to estimate the conditional distributions $P\left(L_{i}|{\tilde{L}}_{i},{\tilde{D}}_{i+1}\right)$ for every *i* and then recover $P\left({\tilde{L}}_{n}|{\tilde{D}}_{n}\right)$ using (8).

### 4.2 Modeling the autoregressive components using conditional normalizing flows

We propose to estimate each autoregressive component $P\left(L_{i}|{\tilde{L}}_{i},{\tilde{D}}_{i+1}\right)$ using a multi-scale conditional normalizing flow architecture. We define a sequence of latent spaces ${\tilde{Z}}_{i+1}≔\left[Z_{i}^{i},\ldots ,Z_{0}^{i}\right]$ of decreasing dimension and a parametrized transformation

$$G_{i}^{\theta }:{\tilde{Z}}_{i+1}\times {\tilde{D}}_{i+1}\times {\tilde{L}}_{i}\to L_{i}.$$

**Figure 2:**
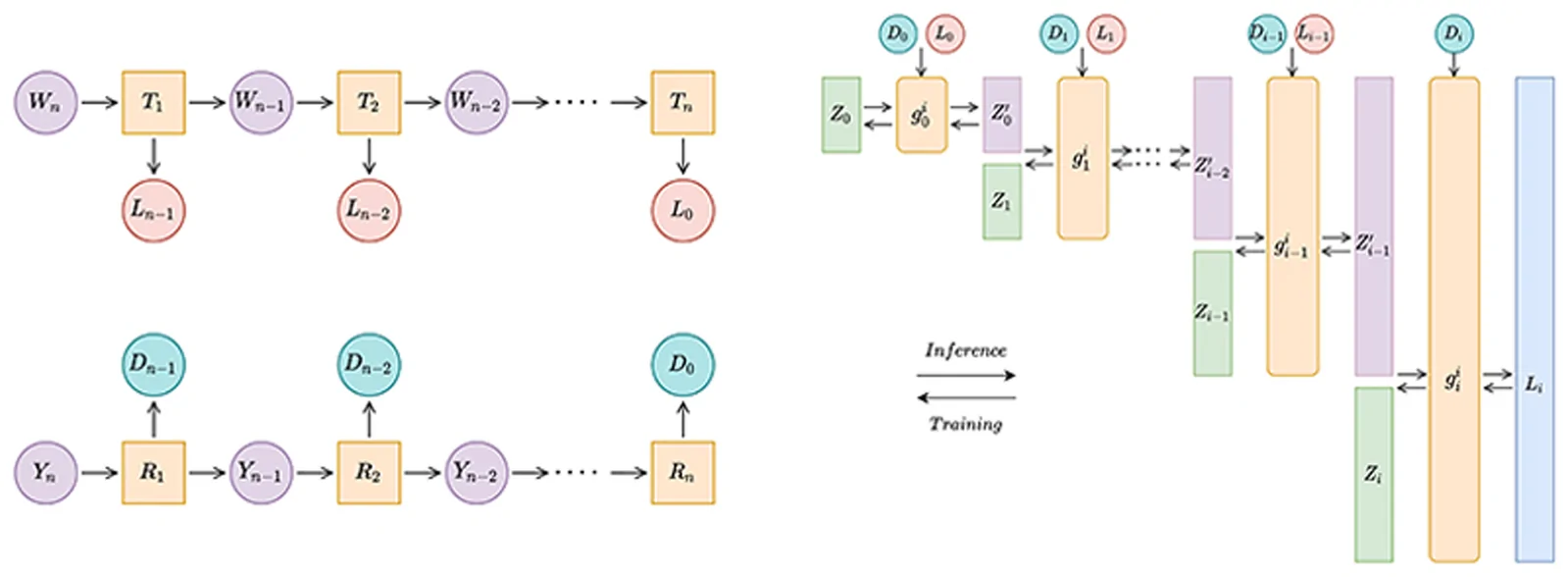
Left: unconditional normalizing flow architecture used to encode conditioning and conditioned images, denoted by *Y*_*n*_ = *Y* and *W*_*n*_ = *W* respectively, into a sequence of hierarchical latent spaces. Right: design of the conditional transformation $G_{i}^{\theta }$ that models the *i*^*th*^ autoregressive component. The index of the flow *i* is omitted in both the transformed latent spaces *Z*_*j*_ and the intermediate latent spaces $Z_{j}^{\prime }$ for simplicity.

The transformations $G_{i}^{\theta }$ are constructed by assembling multi-scale transformations ${\left(g_{j}^{i}\right)}_{j=0}^{i}$ defined as follows:

$$\begin{array}{l} g_{0}^{i}:Z_{0}^{i}\times D_{0}\times L_{0}\to Z_{0}^{\prime i}\\ g_{j}^{i}:\left[Z_{j-1}^{\prime i},Z_{j}^{i}\right]\times D_{j}\times L_{j}\to Z_{j}^{\prime i}\;\text{for}\;j=1,\ldots ,i-1\\ g_{i}^{i}:\left[Z_{i-1}^{\prime i},Z_{i}^{i}\right]\times D_{i}\to L_{i} \end{array}$$

where $Z_{i-1}^{\prime i},\ldots ,Z_{0}^{\prime i}$ are intermediate latent spaces of decreasing dimension, $g_{j}^{i}$ is invertible as a function from $\left[Z_{j-1}^{\prime i},Z_{j}^{i}\right]\;$ to $Z_{j}^{\prime i}$ and $g_{0}^{i}$ is invertible as function from $Z_{0}^{i}$ to $Z_{0}^{\prime i}$. The transformation $G_{i}^{\theta }$ is then obtained by composing the functions $g_{j}^{i}$ in the following way. Given the conditioning variables ${\left(d_{j}\right)}_{j=0}^{i}\in {\tilde{D}}_{i+1}$ and ${\left(l_{j}\right)}_{j=0}^{i-1}\in {\tilde{L}}_{i}$ a latent variable $z_{0}\in Z_{0}^{i}$ is transformed to $z_{0}^{\prime }\in Z_{0}^{\prime i}$ by function $g_{0}^{i}\left(\cdot ;d_{0},l_{0}\right)$. Then $z_{0}^{\prime }$ and ***z***_1_ are concatenated and inserted to $g_{1}^{i}\left(\cdot ;d_{1},l_{1}\right)$ which outputs $z_{1}^{\prime }\in Z_{1}^{\prime i}$. This process continues as implied up until the *i*^*th*^ level whose output is $l_{i}=g_{i}^{i}\left(\left[z_{i-1}^{\prime },z_{i}\right];d_{i}\right)\in L_{i}$. A schematic description of $G_{i}^{\theta }$ is presented in the right diagram of Figure 2.

Denote by $f_{j}^{i}$ the inverse of $g_{j}^{i}$ for fixed conditioning variables in *D*_*j*_ and *L*_*j*_

$$\begin{array}{l} f_{0}^{i}:Z_{0}^{\prime i}\times D_{0}\times L_{0}\to Z_{0}^{i}\\ f_{j}^{i}:Z_{j}^{\prime i}\times D_{j}\times L_{j}\to \left[Z_{j-1}^{\prime i},Z_{j}^{i}\right]\;\text{for}\;j=1,\ldots ,i-1\\ f_{i}^{i}:L_{i}\times D_{i}\to \left[Z_{i-1}^{\prime i},Z_{i}^{i}\right] \end{array}$$

and by $F_{i}^{\theta }$ the inverse of $G_{i}^{\theta }$ as a function from ${\tilde{Z}}_{i+1}$ to *L*_*i*_ obtained by composing the functions $f_{j}^{i}$. We model each $f_{j}^{i}$ by adopting the conditional flow design of Lugmayr et al. [27]. More specifically, we initially use the same squeeze layer, which is followed by two transition steps and *K* conditional flow steps. Each transition step consists of an actnorm layer followed by 1 × 1 invertible convolution layer. Each conditional flow step consists of an actnorm layer followed by 1 × 1 invertible convolution, which is followed by an affine injector and an affine coupling layer. We use from 8 to 16 conditional flow steps depending on the difficulty of the image translation task.

### 4.3 Maximum log-likelihood estimation and training

Here we use the constructed transformations $F_{i}^{\theta }$ to parametrize each autoregressive component $P\left(L_{i}|{\tilde{L}}_{i},{\tilde{D}}_{i+1}\right)$ and, together with the unconditional normalizing flows *R*^*θ*^ and *T*^*θ*^, use them to estimate *P* (*W* |*Y*). With this aim, for every latent space $Z_{j}^{i}$ we choose as prior a multivariate normal distribution, whose density we denote by $𝒩\left(z_{j}^{i};0,I\right)$. Using the Bayes’ rule, the change of variables formula for probability density functions, the factorization in (8) and the chain rule for composition of functions, we parametrize the log-density of the conditional distribution *P* (*W* |*Y*) by

(9) $$\begin{array}{ll} \log p_{W|Y}^{\theta }(w|y) & =\sum_{i=1}^{n}\log\left|\det\frac{\partial T_{i}^{\theta }\left(w_{n-i+1}\right)}{\partial w_{n-i+1}}\right|\\ & +\sum_{i=1}^{n-1}\;\sum_{j=0}^{i-1}\left[\log 𝒩\left(z_{j}^{i};0,I\right)+\log\left|\det\left(\frac{\partial f_{j}^{i}\left(z_{j}^{\prime i};l_{j},d_{j}\right)}{\partial z_{j}^{\prime i}}\right)\right|\right]\\ & +\sum_{i=0}^{n-1}\left[\log 𝒩\left(z_{i}^{i};0,I\right)+\log\left|\det\left(\frac{\partial f_{i}^{i}\left(l_{i};d_{i}\right)}{\partial l_{i}}\right)\right|\right] \end{array}$$

where $l_{i}\in L_{i},{\tilde{l}}_{i}\in {\tilde{L}}_{i}$ and ${\tilde{d}}_{i+1}\in {\tilde{D}}_{i+1}$ implicitly depend on ***w*** and ***y*** through the normalizing flows *R*^*θ*^ and *T*^*θ*^. We refer the reader to Section C in the Appendix for a detailed derivation (9).

We train our CAFLOW model by minimizing the following training objective

$$\log p_{W|Y}^{\theta }(w|y)+\text{λ}\log p_{Y}^{\theta }(y),$$

where $p_{Y}^{\theta }(y)$ is estimated using the unconditional normalizing flow *R*^*θ*^ as in Subsection 2.1. The parameter *λ* acts as a regularizer during training. It can be interpreted as an interpolation parameter between the conditional distribution *P* (*W* |*Y*) (when *λ* = 0) and the joint distribution *P* (*Y, W*) (when *λ* = 1).

### 4.4 Inference

The standard way to perform inference using a trained CAFLOW model is the following:

1. Calculate the conditional encodings $d_{n-1},\ldots ,d_{0}\in {\tilde{D}}_{n}$ by passing the conditioning image through the multi-scale flow *R*^*θ*^.
2. Sample latent variables $z_{i}^{j}$ from 𝒩 (0, *τ* ^2^), where *τ* denotes the sampling temperature.
3. Calculate the output image latent variables $l_{0},\ldots ,l_{n-1}\in {\tilde{L}}_{n}$ by applying the transformations $G_{i}^{\theta }$ sequentially from $G_{0}^{\theta }$ to $G_{n-1}^{\theta }$.
4. Finally, convert the output image latents ***l***_0_, …, ***l***_*n*−1_ to the output image by passing them through the reverse flow (*T*^*θ*^)^−1^.

We have observed that using a sampling temperature *τ* less than 1 typically leads to significant improvement in the quality of the output image at the expense of diversity.

**Figure 3:**
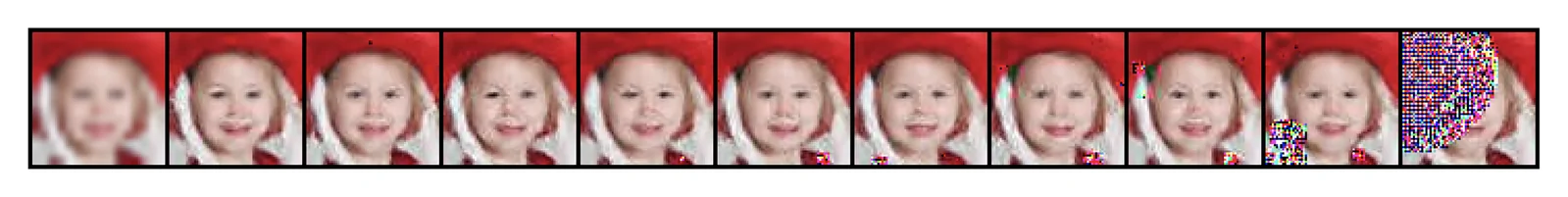
10 super-resolved versions of the LR image in decreasing conditional log-likelihood order.

Our framework can act both as conditional generator and as a conditional likelihood estimator. Figure 3 shows ten super-resolved versions of the low resolution image in decreasing order of conditional log-likelihood. We leverage the conditional likelihood estimation to automatically select the best generated samples and disregard bad samples.

We have observed that CAFLOW typically assigns to the ground-truth image higher conditional log-likelihood than any generated sample. Such observation suggests that CAFLOW is a powerful conditional likelihood estimator, and that better samples can be obtained by the use of an optimisation algorithm, which searches for samples with high conditional likelihood. However, we used a simpler inference method in this work: keep the best *N* out of *M* generated samples based on the conditional log-likelihood. We empirically found that *M* should be larger for higher sampling temperatures.

## 5 Experiments

In this section we evaluate CAFLOW on three image-to-image translation tasks: image super-resolution, image colorization and image inpainting. We refer the reader to section B in the Appendix for more details about the reported experiments and to section E for extended visual results.

### 5.1 Image Super-resolution

**Figure 4:**
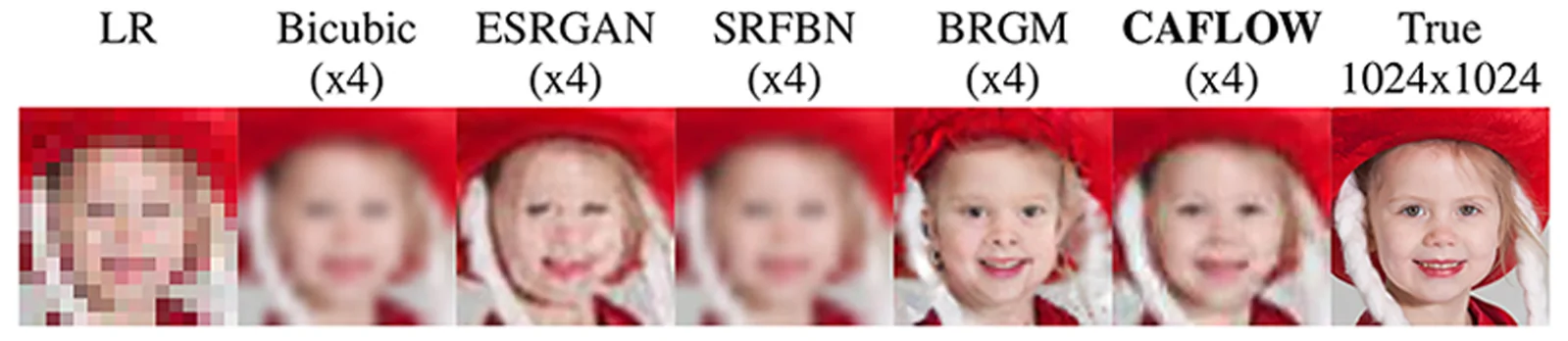
Qualitative evaluation on FFHQ 4x super-resolution of 16x16 resolution images. Left column shows the low resolution input, while the right column shows the true high-quality image. ESRGAN and SRFBN show clear distortion and blurriness. BRGM generates a clear image, but fails to recover the true image. Our method generates the closest image to the ground truth albeit slightly less clear than BRGM.

We evaluate the ability of CAFLOW to perform image super-resolution on the FFHQ dataset [18] (Creative Commons BY-NC-SA 4.0). We resized the original images to 16 × 16 and 64 × 64 patches and trained the model for x4 super-resolution. The quantitative evaluation is performed using the LPIPS and RMSE scores on 100 unseen images. For inference, we used *τ* = 0.5 and kept the sample with the highest conditional log-likelihood out of ten generated samples. A comparison with state-of-the-art methods is shown in Table 1. We present visual results in Figure 4 and refer the reader to the supplementary material for more examples.

Our method outperforms all the other super-resolution methods based on both metrics. It is slightly inferior to BRGM in terms of perceptual quality but it is significantly better in terms of fidelity which is reflected in the quantitative evaluation.

### 5.2 Image Colorization

**Figure 5:**
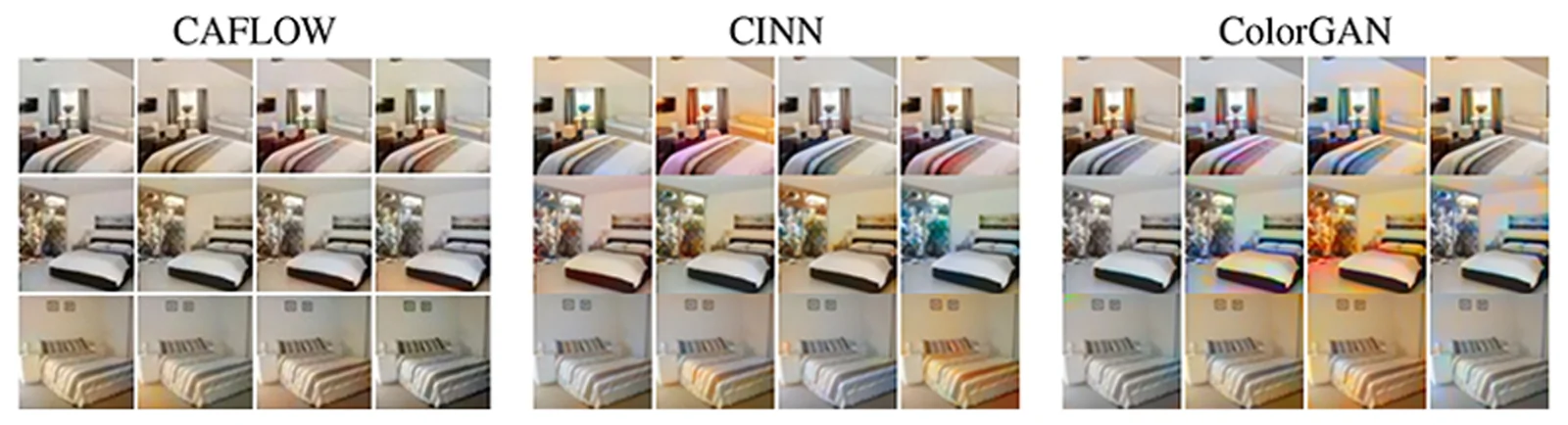
Qualitative evaluation: Four colorizations proposed by CAFLOW, CINN and ColorGAN for three test images. ColorGAN generates unrealistically diverse colorizations with significant color artifacts (for example a yellow region on a white wall). CINN generates more realistic less diverse colorizations with less pronounced color artifacts compared to ColorGAN, which is reflected in the improved FID score. Finally, CAFLOW generates even more realistic and less diverse colorizations than CINN with even rarer color artifacts, which is more representative of the data distribution according to the FID score.

To our knowledge, diverse image colorization has been addressed by conditional flows [2], conditional GANs [5] and recently score-based models [35]. We trained the model on 10% of the LSUN bedroom 64 × 64 training dataset [43], a popular dataset for image colorization. For inference, we used *τ* = 0.85 and kept the best out of ten generated samples for each test image using the conditional log-likelihood. We report the performance of the model in Table 2. We use the FID score to compare our method against the cINN and ColorGAN, which have been trained on the full dataset. We show visual results for all methods in Figure 5. We did not include [35] in the comparison because it was trained on higher resolution images.

Our model outperforms both methods on image colorization based on the FID metric. We calculated the FID score by generating one conditional sample for each test image, so that our evaluation is identical with the evaluation of the other methods. Moreover, we calculated the FID score by generating 5 samples for each test image, which yielded an improved FID score (**16.73**). We suggest that this protocol be adopted by methods which address diverse image colorization in the future.

### 5.3 Image Inpainting

**Figure 6:**
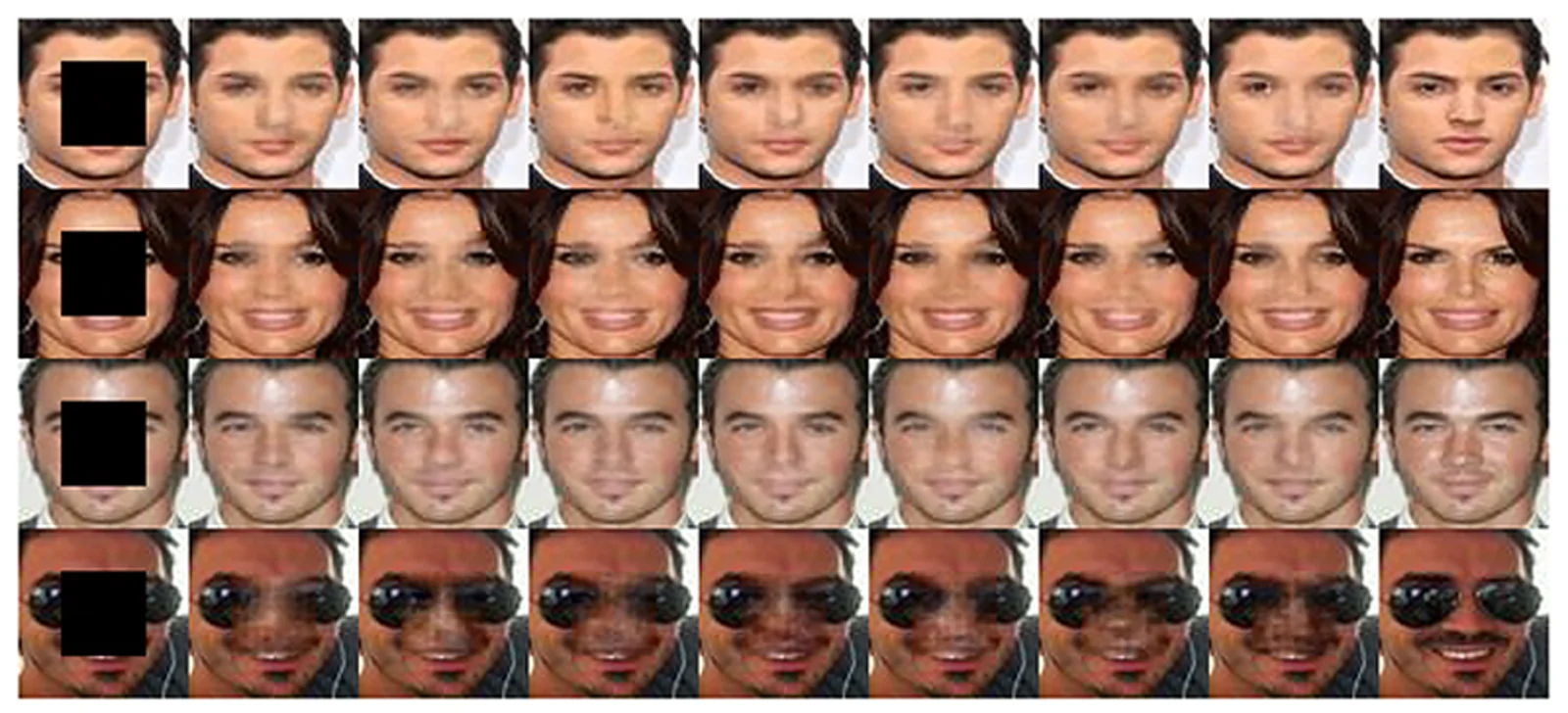
Different inpaintings proposed by CAFLOW with *τ* = 0.5. Ground truth on the right.

We evaluated the performance of CAFLOW on image inpainting by removing central masks covering 25% of the centrally cropped human face of the CelebA dataset [25]. We compare the performance of the model with the conditional flow [26] on the same task using the PSNR metric, see Table 3. We show inpainting examples in Figure 6.

Our model outperforms [26] based on the PSNR metric and on qualititative performance. CAFLOW generates realistic images by blending smoothly the conditioning with the synthesized part of the image in contrast to [26] which generates overly smooth synthesized parts which do not blend well with the surrounding image. Both methods fail in faces which wear sunglasses or face sidewise. We believe that this is attributed to the small number of such training examples.

## 6 Limitations

The main modeling limitation of the framework is attributed to limited expressivity of normalizing flows. Each transformation of a normalizing flow has to be invertible with a tractable calculation of the determinant of the jacobian. This limits the representational power of normalizing flows. However, Wu et al. [41] claim to overcome expressivity limitations of normalizing flows by combining deterministic invertible functions with stochastic sampling blocks. Therefore, incorporating their proposed stochastic blocks in our conditional autoregressive flows can potentially lead to significant performance improvement.

Another limitation of the framework is that it can become computationally expensive for high resolution images where typically more scales are used for the hierarchical latent decomposition of the images. A possible solution to this problem is the use of weight sharing among the conditional autoregressive components as described in section D in the Appendix.

## 7 Conclusion

We have introduced conditional auto-regressive flows, coined CAFLOW, which combine auto-regressive modeling with conditional normalizing flows for image-to-image translation. CAFLOW is an efficient conditional image generator and conditional likelihood estimator able to cross-correlate information at different scales, improving on the expressivity of former conditional flow architectures. We demonstrate its efficiency as conditional generator on standard image-to-image translation tasks such as image super-resolution, image colorization and image inpainting. We find that, in the above-mentioned tasks, CAFLOW achieves better performance than standard conditional flows and conditional GANs. Moreover, we demonstrate its efficiency as conditional likelihood estimator by showing that the model can disregard bad samples and keep the best samples based on the conditional log-likelihood.

## Supplementary Material

Appendices

## Figures and Tables

**Table 1:** Quantitative evaluation of (x4) super-resolution on FFHQ 16^2^. We report LPIPS/RMSE scores for each method. Lower scores are better.

Dataset	CAFLOW	BRGM	ESRGAN	SRFBN	BICUBIC
FFHQ 16^2^	**0.08**/**17.56**	0.24/25.66	0.35/29.32	0.33/22.07	0.34/20.10

**Table 2:** Quantitative evaluation of colorization on LSUN BEDROOM 64 × 64 dataset. We report FID score for each method. Lower scores are better.

Metric	CAFLOW	CINN	ColorGAN
FID 1	**18.15**	26.48	28.31

**Table 3:** Quantitative evaluation of inpainting on the CelebA dataset. We report PSNR and LPIPS scores for each method.

Method	PSNR ↑	LPIPS ↓
**CAFLOW**	**26.08**	0.06
Lu and Huang [26]	24.88	-
