# Supplementary material for: CAFLOW: Conditional Autoregressive Flows
Source: Found Data Sci. Author manuscript; Available in PMC 2026 Oct 16. (PMC7619591; doi:10.3934/fods.2024028)
Supplement: Supplementary file 1 [file EMS218464-supplement-Appendices.pdf]

A Architecture

In this section, we provide more details about the architecture of the unconditional multi-scale flows and the conditional auto-regressive multi-scale flows which comprise our modelling framework shown in Figure 2.

A.1 Unconditional multi-scale flows

The sequence of transformations in each scale are:

1. **Dequantization layer** (only in the first scale). Each pixel of each image channel admits an integer value from 0 to 255. If we do not modify those values, we model a high dimensional distribution in discrete space. However, normalizing flows rely on the rule of change of variables which is naturally defined in continuous space. Therefore, training a normalizing flow on the raw images will result in a model which which will place arbitrarily high likelihood on a few RGB values. This behavior leads to catastrophic destabilization of the training procedure and very poor performance. To remedy this problem, we convert the discrete space to continuous space by dequantizing each pixel value by resampling it from the distribution $q(u_{deq}|u) = \mathcal{U}([u, u+1))$, where $\mathcal{U}([u, u+1))$ is the uniform distribution over the interval $[u, u+1)$. This is called **uniform dequantization** and it is used by the majority of normalizing flow models in the literature. Dequantization effectively represents images as hypercubes. Modelling such sharp borders is challenging for a normalizing flow as it relies on smooth transformations. For this reason, Ho et al. [13] proposed to learn the dequantization distribution $q(x_{deq}|x)$, where x is the quantized and x_{deq} the dequantized image using a conditional normalizing flow. This is called **variational dequantization**. We noticed that using variational dequantization results in a significant increase in performance. For this reason, we decided not to use variational dequantization in the experiments where we compare against other conditional flows, because this would defeat the purpose of fair comparison. We used variational dequantization in the experiments where we compare against methods which do not rely on normalizing flows.
2. **Squeeze layer**. We squeeze the tensor in the channel dimension effectively halving the spatial resolution: the shape of the tensor is transformed from (B, C, H, W) to $(B, 4C, H/2, W/2)$, where B is the batch size, C the number of channels, H the height and W the width of the image.
3. **Two transition steps**. Each transition step consists of an invertible normalization layer (ActNorm) followed by an invertible 1×1 convolution layer. According to [27], the transition step allows the network to learn a linear invertible interpolation between neighboring pixels after the application of the squeeze layer. If the transition steps are not used, the squeeze layer may lead to checkerboard artifacts in the reconstructed image because it is exclusively based on pixel-reordering.
4. **K flow steps**. Each flow step consists of an invertible normalization layer, invertible 1×1 convolution layer and an affine coupling layer in that order. The scale and the bias in the affine coupling layer are computed by a simple convolutional neural network which performs three sequential convolutions with a 3×3, 1×1 and 3×3 convolutional kernels respectively. The number of kernels in each convolutional layer is chosen to be 64.
5. **Split layer**. We split off half of the channels before the squeeze layer of the next scale. Therefore, the next scale transforms only the half part of the tensor. This motivates the modelling of variations in different resolutions and ultimately the hierarchical latent space decomposition of images using multi-scale normalizing flows. Empirically, it has been observed that the first split variables typically encode noise patterns or image details, while the final split variables encode higher level information.

A.2 Conditional multi-scale flows

Each scale of a conditional flow F_i^θ contains the following transformations in order:

1. **Squeeze layer**.
2. **Two transition steps**.
3. **M Conditional flow steps**. We adopt the conditional flow step design used by [27]:
   (a) Invertible normalization (ActNorm).
   (b) Invertible 1×1 convolution.
   (c) Affine injector.
   (d) Conditional affine coupling layer.
4. **Split layer**.

We calculate the scale and the bias of the affine transformations in the affine injector and the conditional affine coupling layer using the same convolutional neural network as the one we used for the unconditional flows. The only difference is that we use 32 instead of 64 kernels.

B Details of experiments

We used the same learning rate scheduling system for all experiments. Initially, we increase the learning rate linearly from 0 to the target learning rate (usually 10^{-3}) in the first 500 iterations. Then, we use the pytorch STEPLR learning rate scheduler with value $\gamma = 0.999$. When the training curve levels-off (around 70K iterations), we reduce the learning rate by a factor of 10 until final convergence. This reduction provides a final performance boost. We do not repeat that reduction because it only results in overfitting.

Moreover, we used exponential moving average (EMA) with a rate equal to 0.999 in all experiments. We empirically found that EMA consistently provides a small increase in performance. Finally, we clipped the norm of the computed gradients to 1, because it improved training stability with no noticeable compromise on performance.

B.1 Image super-resolution

We used 3 scales, 16 flow steps in each level of the conditioning flow R^θ, 32 flow steps in the each level of the conditioned flow T^θ and 12 conditional flow steps in each level of each conditional flow F_i^θ. We set the regularization constant $\lambda = 0.01$. Moreover, we used variational dequantization implemented with a conditional flow of 4 conditional flow steps, because we compared our method against methods which are not based on flows. We trained our model for 4.5 days on a single NVIDIA TESLA P100 GPU on 68K images of the FFHQ dataset with a target learning rate of 10^{-3}. We used 1000 images for validation and 100 images for testing.

B.2 Image colorization

We used 3 scales, 24 flow steps in each level of the conditioning flow R^θ, 24 flow steps in the each level of the conditioned flow T^θ and 12 conditional flow steps in each level of each conditional flow F_i^θ. We set the regularization constant $\lambda = 0.01$. We used uniform dequantization as we intended to compare our method with [2], which is a conditional flow. We trained the model on 300K images of the lsun bedroom dataset (this accounts for 10% of the full dataset) on a single NVIDIA TESLA P100 GPU for 3 days with a target learning rate of 10^{-3}. We used 1000 validation images and 5000 test images.

B.3 Image inpainting

We used 3 scales, 30 flow steps in each level of the conditioning flow R^θ, 30 flow steps in the each level of the conditioned flow T^θ and 16 conditional flow steps in each level of each conditional flow F_i^θ. We set the regularization constant $\lambda = 0.05$ and the target learning rate to 10^{-4}, because we faced stability issues with smaller values of λ and greater values of the target learning rate. We trained

the model on 195K images of the CelebA dataset for 5 days on a single NVIDIA TESLA P100 GPU. We used 1000 images for validation and 2000 images for testing. We used the same preprocessing as Lu and Huang [26] and uniform dequantization.

C Derivations

In this section we provide a detailed derivation of (9), which is part of the training objective.

$$
\begin{aligned}
\log p^{\theta}_{W|Y}(\boldsymbol{w}|\boldsymbol{y}) &= \log\left|\det\left(\frac{\partial T^{\theta}(\boldsymbol{w})}{\partial \boldsymbol{w}}\right)\right| + \log p^{\theta}_{\tilde{L}_n|\tilde{D}_n}(\tilde{\boldsymbol{l}}_n|\widetilde{\boldsymbol{d}}_n) \\
&= \sum_{i=1}^{n} \log\left|\det \frac{\partial T_i^{\theta}(\boldsymbol{w}_{n-i+1})}{\partial \boldsymbol{w}_{n-i+1}}\right| + \log \prod_{i=0}^{n-1} p^{\theta}_{L_i|\tilde{L}_i,\tilde{D}_{i+1}}(\boldsymbol{l}_i|\tilde{\boldsymbol{l}}_i, \widetilde{\boldsymbol{d}}_{i+1}) \\
&= \sum_{i=1}^{n} \log\left|\det \frac{\partial T_i^{\theta}(\boldsymbol{w}_{n-i+1})}{\partial \boldsymbol{w}_{n-i+1}}\right| + \sum_{i=0}^{n-1} \log p^{\theta}_{L_i|\tilde{L}_i,\tilde{D}_{i+1}}(\boldsymbol{l}_i|\tilde{\boldsymbol{l}}_i, \widetilde{\boldsymbol{d}}_{i+1}).
\end{aligned}
$$

The justification for the first line can be found in Section 3 of [37]. We go from the first line to the second using the chain rule for the first term and by factorizing the conditional distribution under the dependency assumptions of our model for the second term. The calculation of the first term is straightforward, because we have chosen invertible components with a tractable calculation of the determinant of the Jacobian. We name the second term B for further analysis.

$$
\begin{aligned}
B &= \sum_{i=0}^{n-1} \log p^{\theta}_{L_i|\tilde{L}_i,\tilde{D}_{i+1}}(\boldsymbol{l}_i|\tilde{\boldsymbol{l}}_i, \widetilde{\boldsymbol{d}}_{i+1}) \\
&= \sum_{i=0}^{n-1} \log p_{\tilde{Z}_{i+1}}(F_i^{\theta}(\boldsymbol{l}_i;\tilde{\boldsymbol{l}}_i, \widetilde{\boldsymbol{d}}_{i+1})) + \log\left|\det \frac{\partial F_i^{\theta}(\boldsymbol{l}_i;\tilde{\boldsymbol{l}}_i, \widetilde{\boldsymbol{d}}_{i+1})}{\partial \boldsymbol{l}_i}\right| \\
&= \sum_{i=1}^{n-1}\sum_{j=0}^{i-1}\left[\log \mathcal{N}(\boldsymbol{z}_j^i; \mathbf{0}, \mathbf{I}) + \log\left|\det\left(\frac{\partial f_j^i(\boldsymbol{z}'^i_j; \boldsymbol{l}_j, \boldsymbol{d}_j)}{\partial \boldsymbol{z}'^i_j}\right)\right|\right] \\
&\quad + \sum_{i=0}^{n-1}\left[\log \mathcal{N}(\boldsymbol{z}_i^i; \mathbf{0}, \mathbf{I}) + \log\left|\det\left(\frac{\partial f_i^i(\boldsymbol{l}_i; \boldsymbol{d}_i)}{\partial \boldsymbol{l}_i}\right)\right|\right]
\end{aligned}
$$

The second line is simply obtained by using the change of variables formula. We go from the second line to the third by using the chain rule for the composition of functions (remember the definition of F_i^{θ} in 4.2) and the assumption that the latent variables $Z_i^i, Z_{i-1}^i, ..., Z_0^i$ which comprise $\tilde{Z}_{i+1}$ are i.i.d. with distribution $\mathcal{N}(\mathbf{0}, \mathbf{I})$. The sum is broken into two sums, because the f_i^i functions are conditioned only on $\boldsymbol{d}_i$. Therefore,

$$
\begin{aligned}
\log p^{\theta}_{W|Y}(\boldsymbol{w}|\boldsymbol{y}) = &\sum_{i=1}^{n} \log\left|\det \frac{\partial T_i^{\theta}(\boldsymbol{w}_{n-i+1})}{\partial \boldsymbol{w}_{n-i+1}}\right| \\
&+ \sum_{i=1}^{n-1}\sum_{j=0}^{i-1}\left[\log \mathcal{N}(\boldsymbol{z}_j^i; \mathbf{0}, \mathbf{I}) + \log\left|\det\left(\frac{\partial f_j^i(\boldsymbol{z}'^i_j; \boldsymbol{l}_j, \boldsymbol{d}_j)}{\partial \boldsymbol{z}'^i_j}\right)\right|\right] \\
&+ \sum_{i=0}^{n-1}\left[\log \mathcal{N}(\boldsymbol{z}_i^i; \mathbf{0}, \mathbf{I}) + \log\left|\det\left(\frac{\partial f_i^i(\boldsymbol{l}_i; \boldsymbol{d}_i)}{\partial \boldsymbol{l}_i}\right)\right|\right]
\end{aligned}
$$

D Weight sharing

We mentioned in the second paragraph of Section 6 that the framework can become computationally expensive for modelling conditional distributions of high resolution images, because more scales are needed for the latent decomposition of those images. We claimed that a possible solution to this is the sharing of certain weights between conditional auto-regressive flows of different levels. In particular, we propose that the weights of the networks $f_k^{k+1}, f_k^{k+2}, ...f_k^{n-1}$ are shared for all k satisfying $0 \leq k \leq n-2$. To make this clearer, we present two versions of the framework with four scales in Figure 7: one with no sharing and one where we highlight the shared functions with the same color. Our choice to use the same weights for those functions is based on the fact that those functions share the same conditions and also operate on activations of the same shape at the same level down the flows.

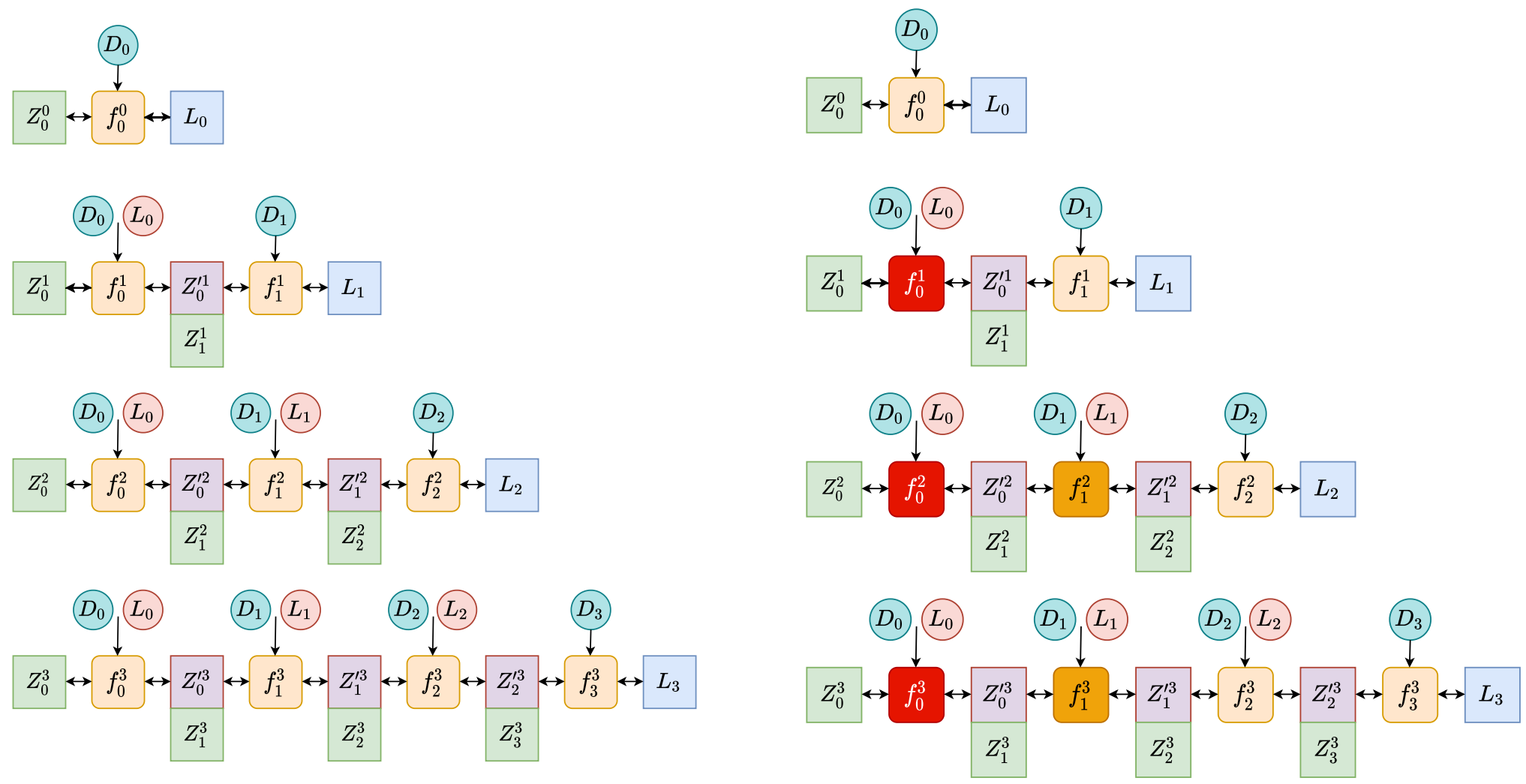

Figure 7: Conditional auto-regressive framework with four scales. Left: No weight sharing. Total number of functions: $n(n+1)/2$ where n the number of scales. Right: Weight sharing (functions with the same vivid color share weights). Total number of functions: $2n-1$.

We experimented with this modification for low resolution images (64×64 images), because we intended to compare it with the theoretical approach. We found that it can yield good performance albeit slightly inferior to the performance of the theoretical framework. Given that this modification reduces the number of internal f_i^j functions from quadratic to linear and still performs well, we believe that it could be used successfully for modelling conditional distributions of high resolution images where more scales/levels n are typically needed. We intend to explore this direction further in the future.

E Visual results

E.1 Image super-resolution

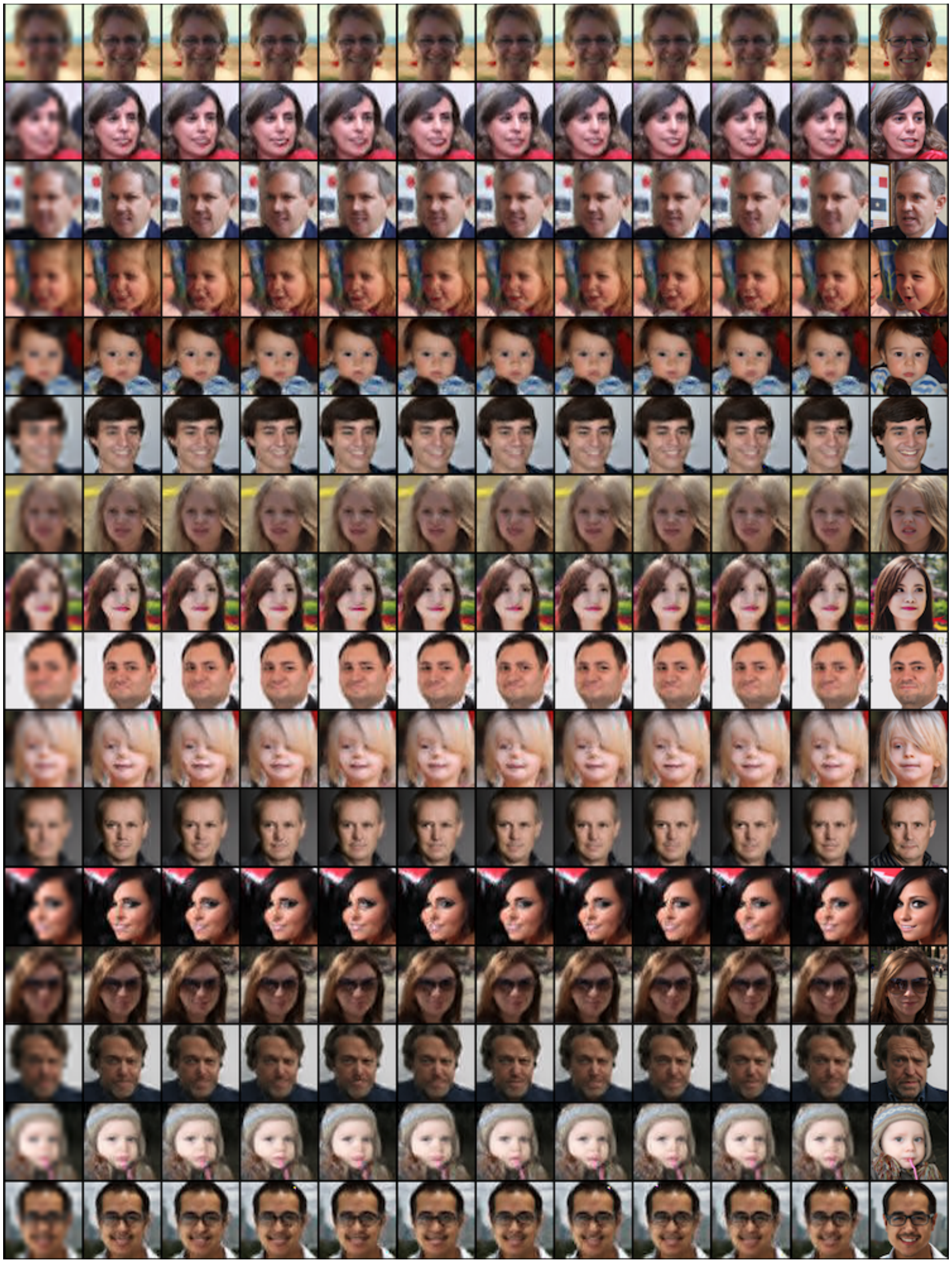

Figure 8: Image super-resolution on the FFHQ dataset. Left: LR bicubicly upsampled. Right: HR image. Middle: 10 super-resolved versions in decreasing conditional log-likelihood order from left to right. We sampled 20 super-resolved images for each LR image and we present the 10 images with the highest conditional log-likelihood. We used sampling temperature $\tau = 0.5$.

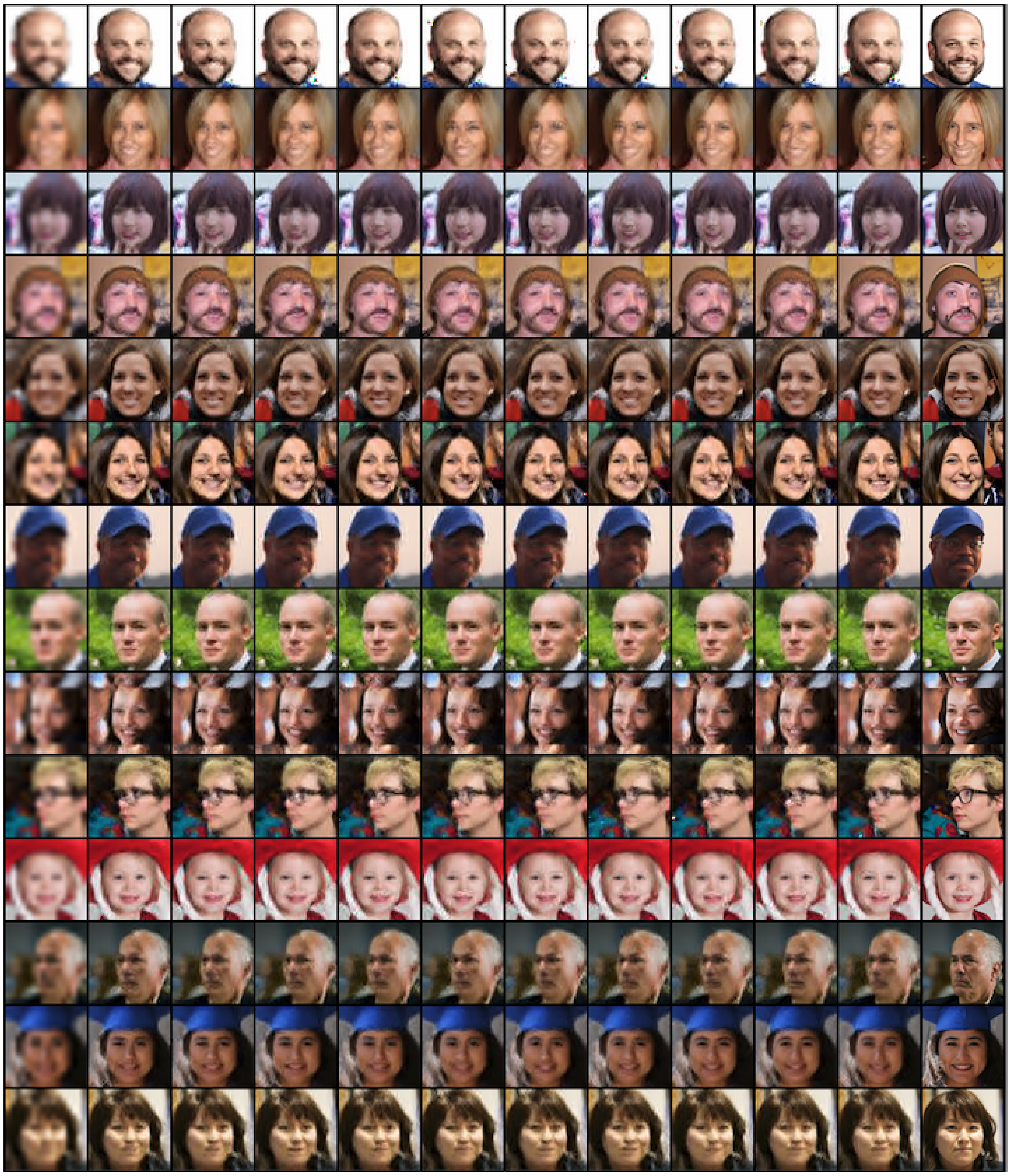

Figure 9: Image super-resolution on the FFHQ dataset. Left: LR bicubicly upsampled. Right: HR image. Middle: 10 super-resolved versions in decreasing conditional log-likelihood order from left to right. We sampled 20 super-resolved images for each LR image and we present the 10 images with the highest conditional log-likelihood. We used sampling temperature $\tau = 0.55$.

E.2 Image inpainting

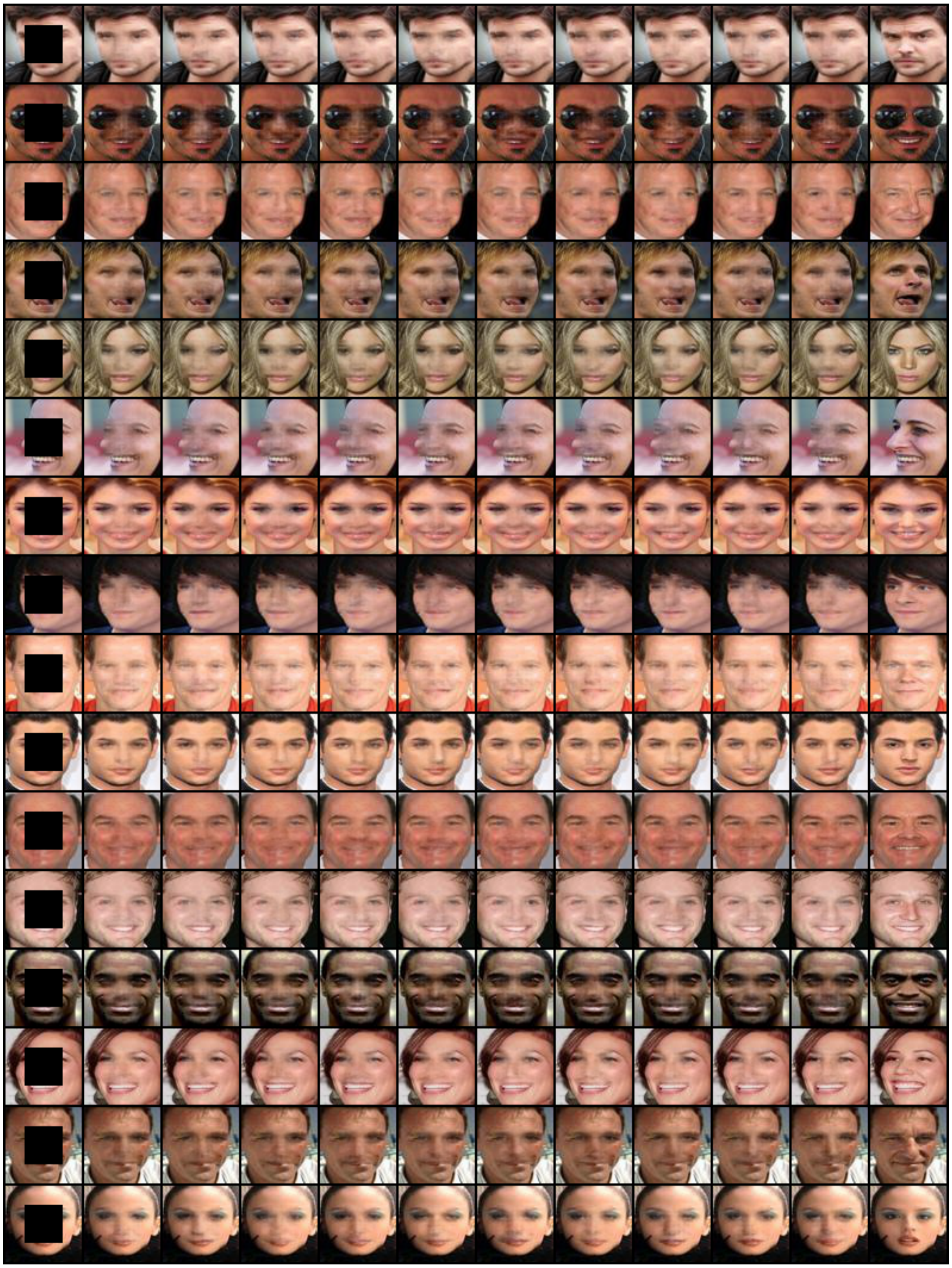

Figure 10: Image inpainting on the CelebA dataset. Left: Masked image. Right: Ground truth. Middle: 10 inpainted versions in decreasing conditional log-likelihood order from left to right. We sampled 30 inpainted images for each masked image and we present the 10 images with the highest conditional log-likelihood. We used sampling temperature $\tau = 0.5$.

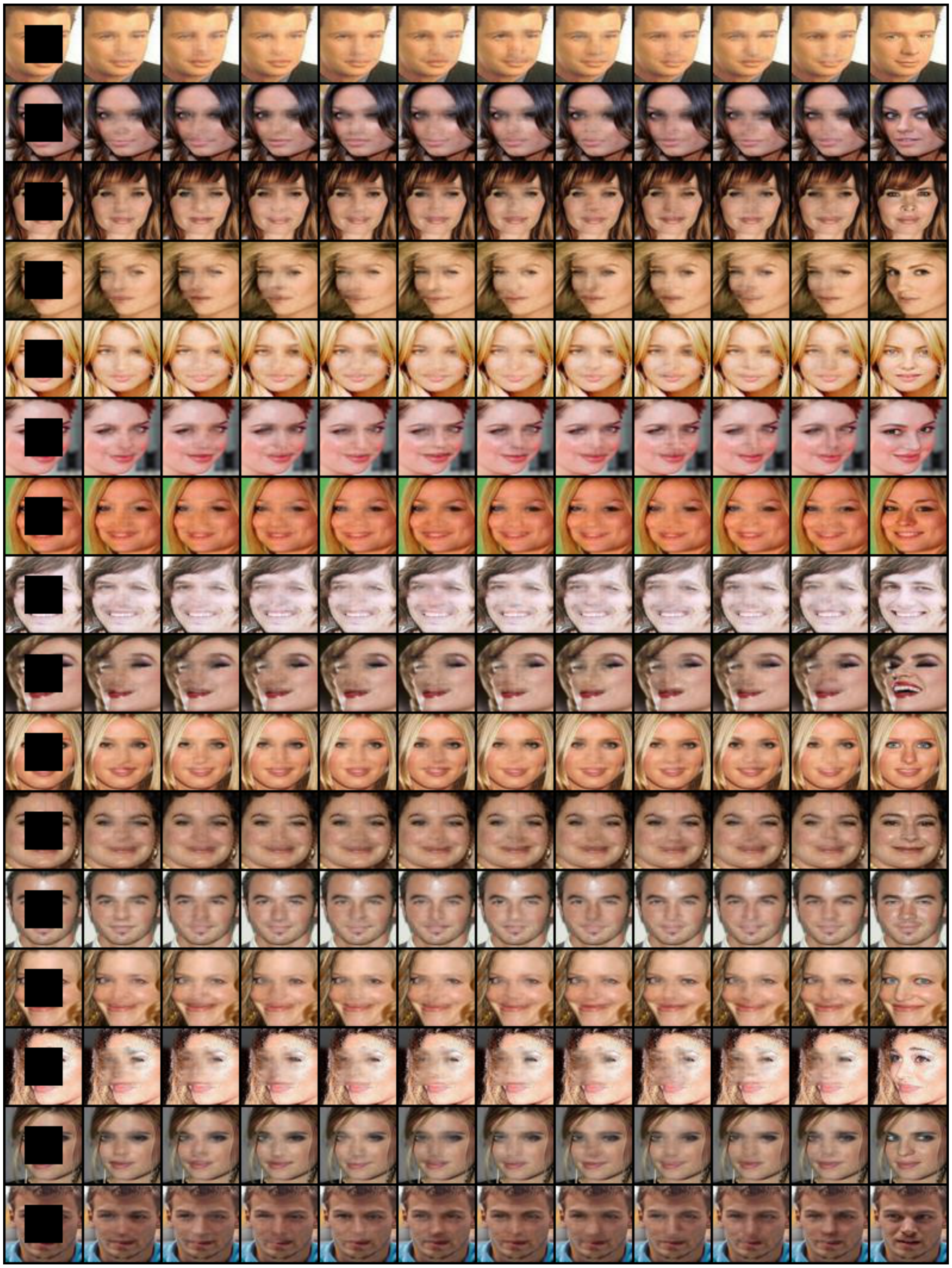

Figure 11: Image inpainting on the CelebA dataset. Left: Masked image. Right: Ground truth. Middle: 10 inpainted versions in decreasing conditional log-likelihood order from left to right. We sampled 30 inpainted images for each masked image and we present the 10 images with the highest conditional log-likelihood. We used sampling temperature $\tau = 0.5$.

E.3 Image colorization

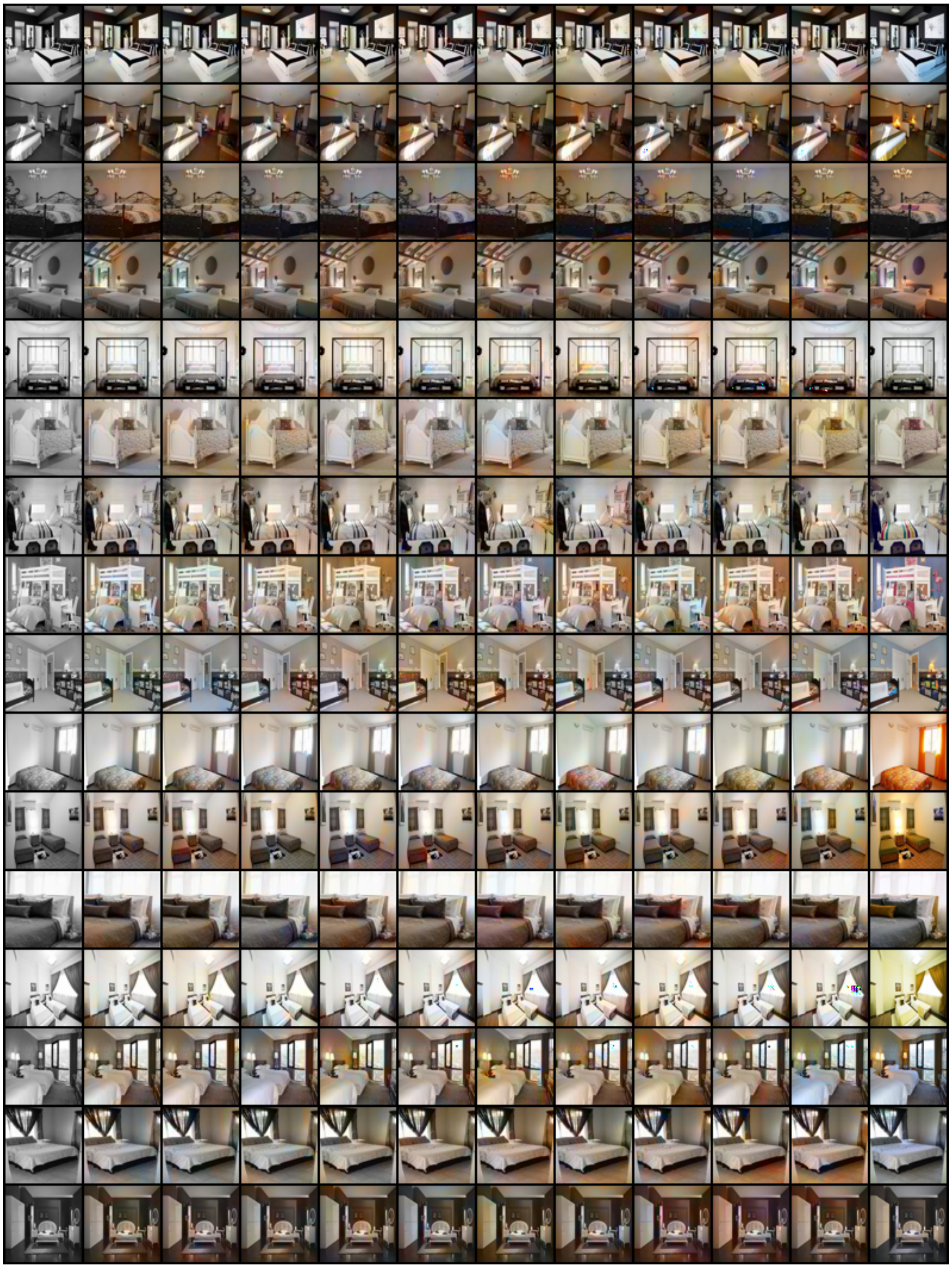

Figure 12: Image colorization on the LSUN BEDROOM dataset. Left: Grayscale image. Right: Ground truth. Middle: 10 colorized versions in decreasing conditional log-likelihood order from left to right. We sampled 25 colorized images for each greyscale image and we present the 10 images with the highest conditional log-likelihood. We used sampling temperature $\tau = 0.85$.

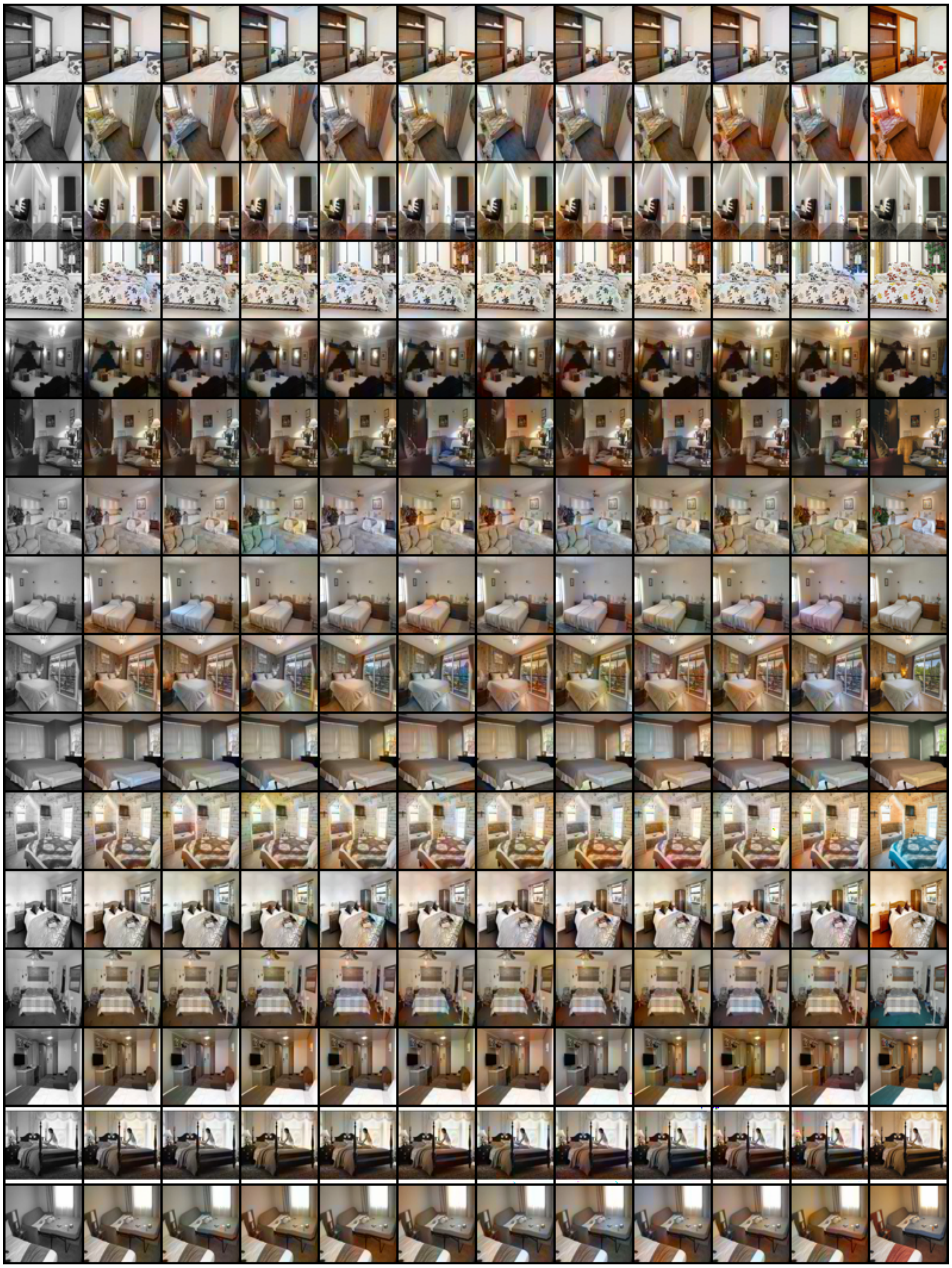

Figure 13: Image colorization on the LSUN BEDROOM dataset. Left: Grayscale image. Right: Ground truth. Middle: 10 colorized versions in decreasing conditional log-likelihood order from left to right. We sampled 25 colorized images for each greyscale image and we present the 10 images with the highest conditional log-likelihood. We used sampling temperature $\tau = 0.85$.

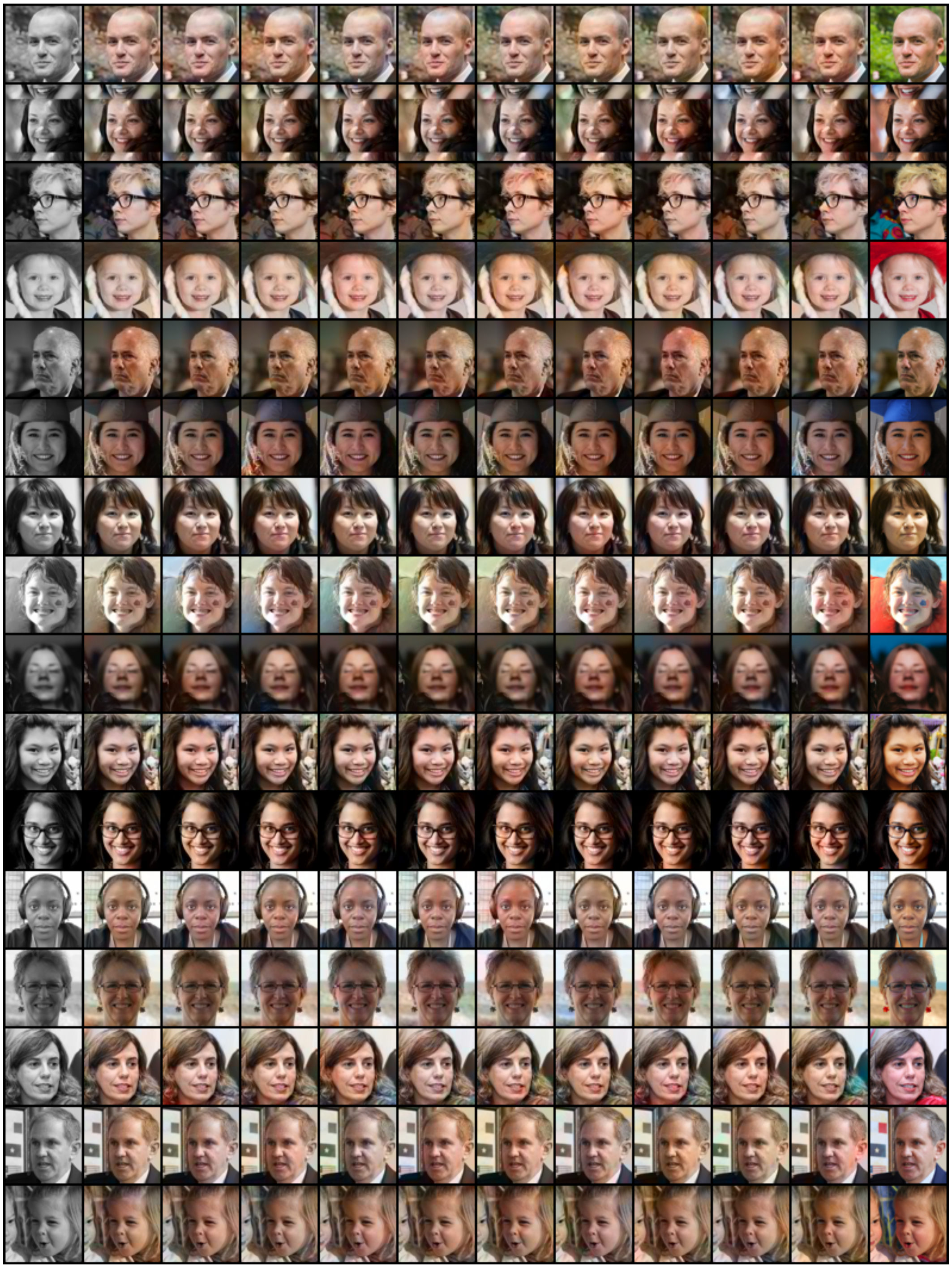

Figure 14: Image colorization on the FFHQ dataset. Left: Grayscale image. Right: Ground truth. Middle: 10 colorized versions in decreasing conditional log-likelihood order from left to right. We sampled 25 colorized images for each greyscale image and we present the 10 images with the highest conditional log-likelihood. We used sampling temperature $\tau = 0.7$.

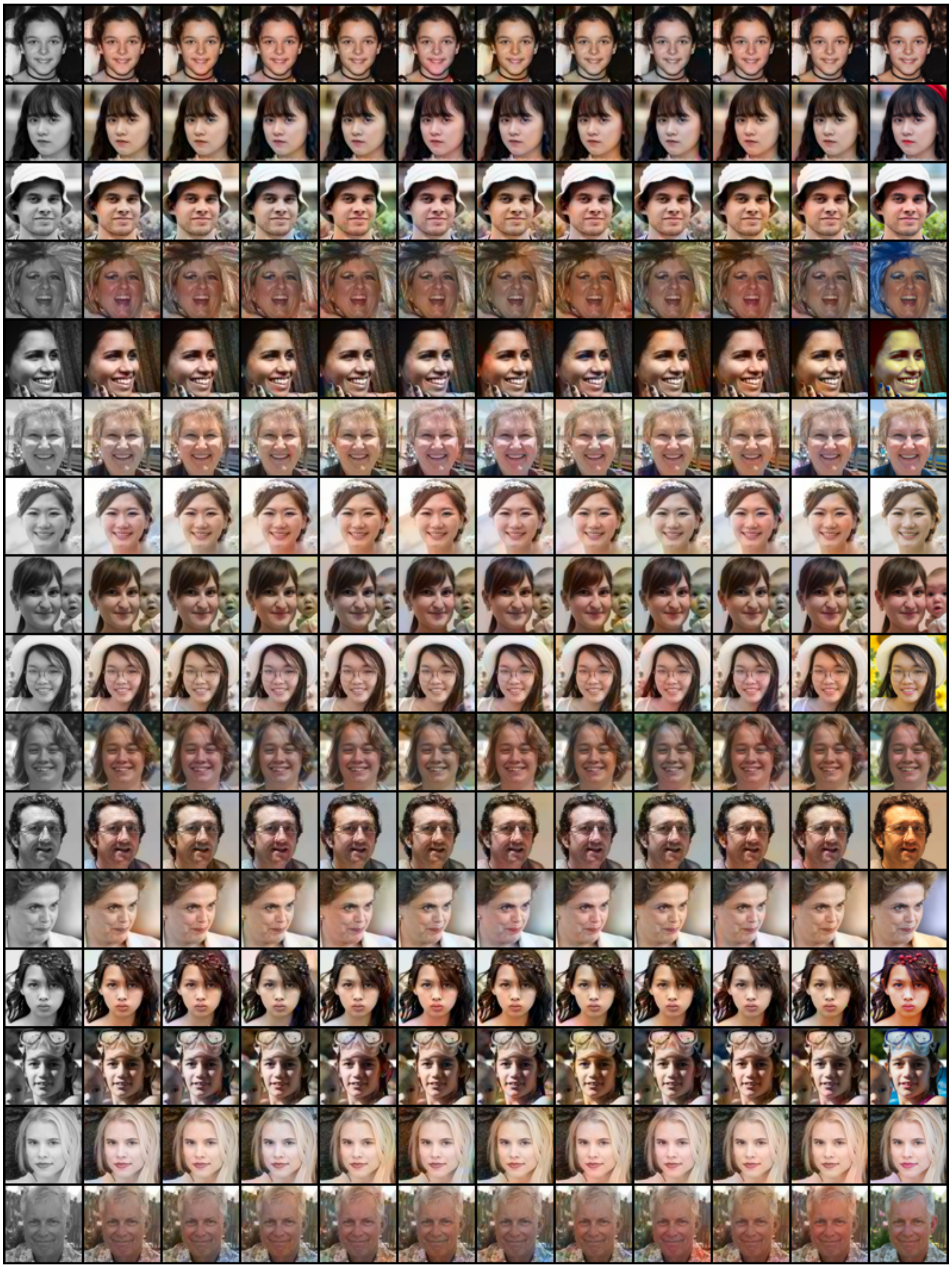

Figure 15: Image colorization on the FFHQ dataset. Left: Grayscale image. Right: Ground truth. Middle: 10 colorized versions in decreasing conditional log-likelihood order from left to right. We sampled 25 colorized images for each greyscale image and we present the 10 images with the highest conditional log-likelihood. We used sampling temperature $\tau = 0.7$.

E.4 Sketch to image synthesis

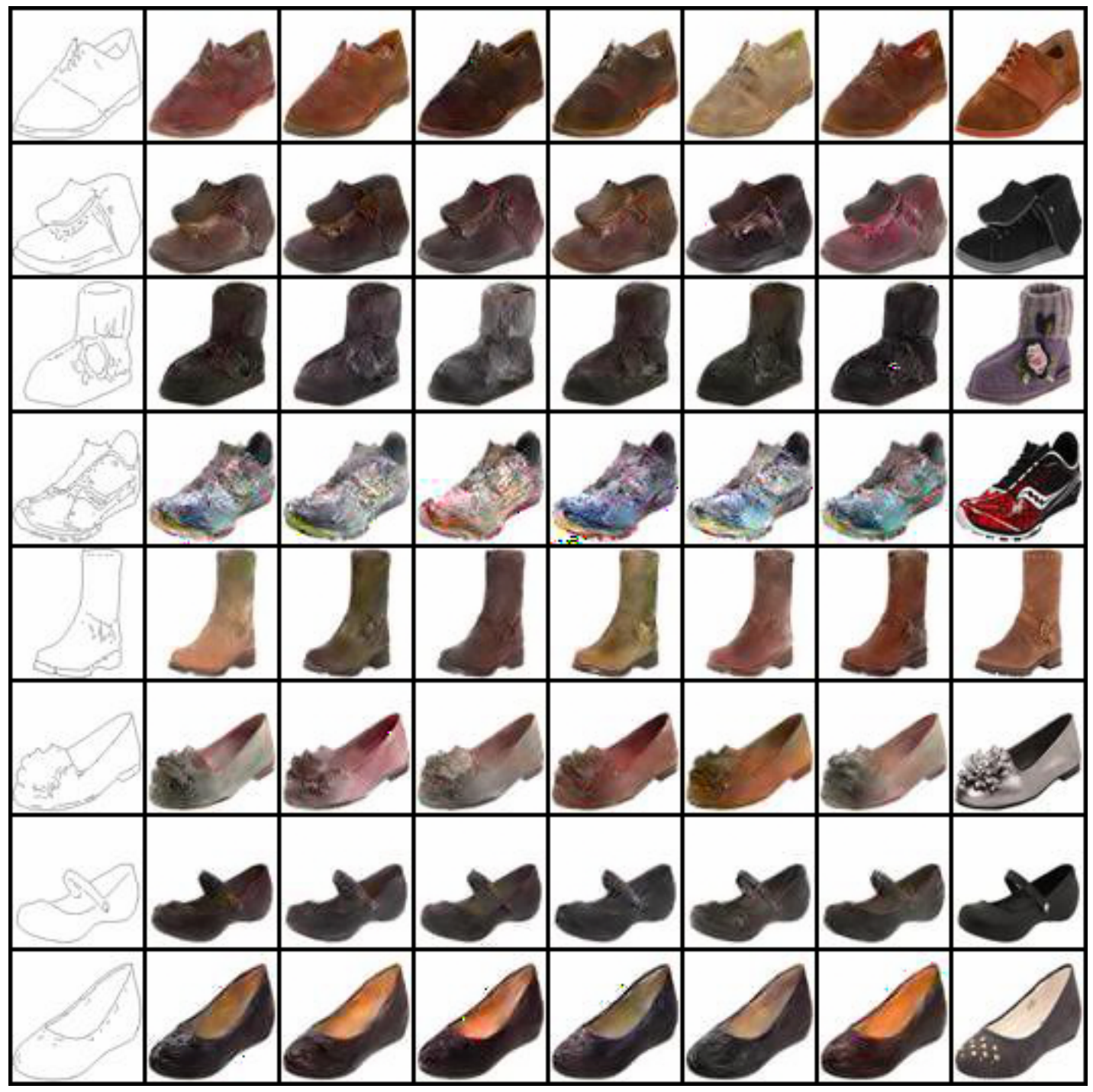

Figure 16: Sketch to image synthesis on the edges2shoes dataset [17]. Left: Sketch. Right: Ground truth. Middle: 6 samples taken with sampling temperature $\tau = 0.8$.
